# Repair and Strengthening of Existing Reinforced Concrete Structures: Recent Advances and Developments

**DOI:** 10.3390/ma19091807

**Published:** 2026-04-29

**Authors:** Andreas Lampropoulos

**Affiliations:** Laboratory of Reinforced Concrete, School of Civil Engineering, National Technical University of Athens (NTUA), 15772 Athens, Greece; andlampropoulos@mail.ntua.gr

Recent devastating earthquakes have highlighted the urgent need for structural upgrades to existing Reinforced Concrete (RC) structures that are vulnerable to failure and collapse. At the same time, the climate emergency requires immediate action, emphasizing the importance of developing cost-effective and sustainable solutions that enhance both the structural performance and energy efficiency of existing buildings.

RC is the most widely used composite material in civil engineering applications. However, the majority of the existing RC structures in earthquake-prone areas require strengthening, as they were often designed according to outdated code provisions or have been damaged due to extreme loading conditions. One of the traditional methods for improving the structural condition of deficient RC structures is the use of conventional concrete, such as the addition of RC layers or jackets [1,2,3,4,5,6,7,8,9]. This technique involves constructing an additional RC element connected to the existing structure, aiming to enhance structural performance and ductility. Although this method has proven effective, special attention must be given to the connection between the new and existing elements. Furthermore, factors such as concrete shrinkage and the preloading of the original structure significantly influence the performance of the strengthened elements and must be carefully considered during analysis and design [3,4,7,8,9].

Ultra-High-Performance Fibre-Reinforced Concrete (UHPFRC) is a material that exhibits superior mechanical properties. In recent years, there has been growing interest in the development of UHPFRC with enhanced characteristics for structural engineering applications [10,11,12,13,14,15,16], particularly in cases involving extreme loading conditions, such as earthquakes, and in thin structural elements like slabs and bridge decks. UHPFRC has also proven highly effective for the strengthening of existing structures [17,18,19,20,21,22,23,24], as it can significantly improve stiffness, flexural strength, and ductility. Previous studies have shown that combining UHPFRC with steel reinforcement leads to superior mechanical performance [19,21,22,23,24]. UHPFRC layers reinforced with steel bars can therefore be widely applied for strengthening existing structures. Comparative studies between conventional RC and UHPFRC strengthening techniques indicate that UHPFRC jackets or layers, combined with steel reinforcement, provide the most effective solution, achieving substantial improvements in stiffness and ultimate load capacity [23,24]. These studies show the great potential of UHPFRC as a strengthening material, which can be particularly effective in specialized strengthening applications including thin overlays for bridge decks, seismic strengthening of columns and beam–column joints, and structures subjected to extreme loading conditions, fatigue, impact, or aggressive environment.

In this context, recent studies [25,26,27,28] have focused on the development of innovative repair and strengthening techniques using both conventional and advanced materials. Experimental research on high-strength grouts incorporating coral sand has emphasized the importance of controlling early-age shrinkage, a critical parameter that may adversely affect structural performance [25]. Various mix designs have been investigated, examining the influence of early strength agents, plastic expansive agents, and post-hardening expansive agents, as well as their combined effect, in order to effectively regulate shrinkage behaviour [25]. Rapid strengthening of RC beams can also be achieved through external prestressing techniques. Different configurations of externally prestressed steel reinforcement have been investigated to optimize improvements in flexural capacity [26]. In addition, Fibre-Reinforced Polymers (FRPs) have emerged as an efficient strengthening solution due to their high strength-to-weight ratio, durability, and ease of installation. However, the fatigue performance of FRP-strengthened elements remains a critical consideration. To address this, soft computing approaches and machine learning models have been developed to improve the prediction accuracy of fatigue life and enhance structural performance assessments [27]. Furthermore, interpretable machine learning techniques have been applied to develop predictive models for concrete cover separation failure modes. These models have been benchmarked against existing design codes and previous research, demonstrating improved predictive capability and offering valuable insights for design and assessment practices [28].

Overall, advancements in materials, combined with the integration of emerging technologies, are expected to play a crucial role in shaping the next generation of resilient and sustainable structural systems. These developments will contribute to safer built environments and reduce the impact of natural hazards. Future research is anticipated to focus on the development of innovative strengthening solutions that enable rapid and efficient implementation while providing enhanced mechanical performance, improved durability, and long-term reliability. Furthermore, these approaches may be integrated with appropriate systems aimed at improving energy efficiency, thereby promoting more sustainable and multifunctional retrofit strategies. At the same time, significant progress is anticipated in advanced numerical and analytical frameworks, incorporating data-driven and machine learning approaches, to enable more accurate prediction of complex failure mechanisms and to support performance-based design and assessment of strengthened structures. Finally, further numerical and experimental research is required, alongside the establishment of comprehensive design standards, to enable the wider adoption of these innovative materials and strengthening techniques.
